# Xanthan Gum as an Eco-Friendly Corrosion Inhibitor for N80 Carbon Steel Under High Pressure and High Temperature in Saline CO_2_-Saturated Solution

**DOI:** 10.3390/ma18194450

**Published:** 2025-09-23

**Authors:** Gaetano Palumbo

**Affiliations:** AGH University of Krakow, Faculty of Foundry Engineering, Department of Chemistry and Corrosion of Metals, al. A. Mickiewicza 30, 30-059 Krakow, Poland; gpalumbo@agh.edu.pl

**Keywords:** xanthan gum, CO_2_ corrosion, corrosion inhibition, carbon steel N80, high temperature and high pressure

## Abstract

In this study, polysaccharide xanthan gum (XG), used in the oil and gas industry as a thickening agent, was evaluated as an active anti-corrosion component against sweet corrosion at high temperatures and pressures in a saline environment for N80 carbon steel in the oil and gas industry. The evaluation involved measuring weight loss and conducting electrochemical assessments at 5 bar CO_2_ partial pressure, different temperatures (e.g., 30 °C and 90 °C), and immersion times (up to 72 h). The electrochemical results indicated that XG effectively mitigated CO_2_ corrosion at both low and high temperatures, demonstrating inhibition efficiencies of 70.10% at 30 °C and 61.41% at 90 °C using 1.0 g L^−1^ of XG, after 24 h. The good inhibition efficiency observed even at high temperatures is likely due to the denaturation process that XG undergoes at high temperatures, where a rigid double-stranded helical structure transitions into two single-stranded, more flexible, worm-like macromolecular conformations. This increases the number and mobility of XG macromolecules available for adsorption on the metal surface. EIS measurements have shown that XG was capable of protecting the metal surface even after prolonged exposure. Potentiodynamic measurements indicated that the inhibitive action of XG is of a mixed type. The Temkin adsorption isotherm model provided a good fit for the observed data, and the calculated parameters suggested that the adsorption of XG primarily occurred through physical adsorption processes, with a contribution from chemical processes. The associated activation energy and the heat of adsorption further supported the physical nature of XG’s adsorption. FTIR analysis was employed to elucidate the interaction between the XG and the N80 carbon steel surface, while SEM-EDS analysis provided visual confirmation of the XG’s impact on the metal surface.

## 1. Introduction

CO_2_ is a significant greenhouse gas that contributes to global warming, primarily released from the burning of fossil fuels, industrial activities, and other sources [1]. One promising solution to mitigate rising CO_2_ levels in the atmosphere is CO_2_-enhanced oil recovery (CO_2_-EOR) [2]. This technique involves injecting CO_2_ into oil reservoirs to enhance oil production [3]. A portion of the injected CO_2_ remains trapped underground, while the rest is cycled back to the surface for reinjection, creating a closed-loop system that supports long-term CO_2_ management storage. Consequently, the crude oil extracted through this method is often distinguished as “carbon-negative oil,” highlighting its potential environmental benefits [4]. However, despite these advantages, the presence of dissolved CO_2_ poses significant challenges to the integrity of steel-based infrastructure in the oil and gas industry [5,6,7,8]. The risk of corrosion increases, particularly in environments with high chloride concentrations, which can damage the materials used in extraction and processing facilities [3,6].

N80 carbon steel is commonly used due to its favorable mechanical properties and cost-effectiveness; however, it is especially susceptible to sweet corrosion, as it cannot form the protective film that characterizes stainless-steel alloys [5,6,7,8]. To address sweet corrosion, two primary strategies are often employed: utilizing high-corrosion-resistant alloys or applying corrosion inhibitors (CIs). While the former can be prohibitively expensive for large-scale reservoir operations, the latter offers a more cost-effective means of reducing the risk of corrosion.

Corrosion inhibitors work by forming an adsorptive layer that serves as a strong barrier, protecting the steel surface from the harsh, corrosive environment. CIs contain heteroatoms and other elements that serve as adsorption centers. These elements form coordination bonds with the metal surface, which can prevent corrosion media from damaging its surface. Currently, the petroleum industry promotes strict compliance with the standards of the Paris Commission (PARCOM) regulation when developing highly effective sweet CIs [3,9]. According to PARCOM, an ideal green inhibitor is non-toxic, readily biodegradable, and does not bioaccumulate. A key criterion is the partition coefficient value (log P_o/w_), which indicates whether the inhibitor molecule is hydrophilic or hydrophobic. PARCOM specifies a standard of “log P_o/w_ < 3” for a non–toxic molecule. Higher log P_o/w_ values suggest increased hydrophobicity, longer environmental persistence, and heightened toxicity [3,9]. A review of the existing literature reveals a common reliance on nitrogen-based compounds, such as various imidazoline and amine derivatives, as CIs in the petroleum industry [2,3,5,9,10]. However, these compounds frequently raise environmental concerns because of their toxicity [3,9]. This has led to a growing interest in investigating naturally occurring CIs that are both efficient and eco-friendly.

Plant extract polymers have been highly regarded as eco-friendly CIs against sweet corrosion. They are plentiful, eco-friendly, and possess good solubility [6,8,11,12,13]. However, when compared to commercial inhibitors, their performance is relatively modest. Umoren et al. [14] compared the corrosion protective abilities of a commercially available corrosion inhibitor with two natural compounds, namely carboxymethyl cellulose and chitosan, in a CO_2_-saturated saline solution. The commercial inhibitor maintained a consistent corrosion inhibition efficiency of approximately 90% at both lower and higher temperatures (e.g., 25 °C and 60 °C). In contrast, the inhibition efficiency of the tested polysaccharides was around 40%.

Drilling fluid (DF) used in the oil and gas industries is generally a water-based chloride solution (e.g., NaCl and/or KCl) that includes various chemical additives such as thickening and crosslinking agents, corrosion and scaling inhibitors, and others. The composition is carefully selected to address specific needs in well drilling, such as wellbore stability, cuttings removal, and minimizing formation damage. When introducing a new component into a drilling fluid formulation, careful consideration is necessary regarding its impact on the overall properties and performance of the fluid. Therefore, replacing commercial inhibitors with natural ones following current regulations, while maintaining the same level of protection and not adversely affecting the overall performance of the DF, can be a challenging task.

Naturally occurring compounds such as guar gum, gum arabic, etc., are often used in DFs as a thickener to enhance the viscosity of the injected water. In recent decades, this research group has studied the anti-corrosion properties of guar gum [8] and gum arabic [6,15], observing that they can mitigate sweet corrosion and act as active components against it.

Xanthan gum (XG) is a natural polysaccharide mainly produced by the bacterium Xanthomonas campestris, known for its strong resistance to acids, bases, salts, and heat [16,17,18]. XG is renowned for its versatility, finding applications in various fields, including biomedical research, wastewater treatment, and food processing [17]. XG is also widely used in DFs as a thickener [19,20,21]. In recent years, evidenced by the growing number of publications, XG has also emerged as a promising, environmentally friendly corrosion inhibitor for metal infrastructures. Arukalam et al. tested XG as a potential corrosion inhibitor for aluminum in a 1.0 M HCl solution, revealing an inhibition efficiency (IE) of around 80.57% at 60 °C [22]. Mobin et al. investigated the effect of XG for mild steel in 1 M HCl at various temperatures, observing a good IE at both low and high temperatures (e.g., 74.24% and 55.40% at 30 °C and 60 °C, respectively) [18]. Following this study, a wealth of literature has emerged, affirming XG’s effectiveness as a corrosion inhibitor across various materials and environments [16,17,23,24,25,26].

In line with the commitment to researching sustainable corrosion inhibitors that mitigate sweet corrosion, this study presents, for the first time, the corrosion-inhibition capabilities of xanthan gum as an environmentally friendly alternative for protecting N80 carbon steel in CO_2_-saturated saline solutions at different temperatures, relevant to the oil and gas industry. All previous studies have focused on XG as a corrosion inhibitor in strong acid solutions, such as HCl and H_2_SO_4_, on different grades of carbon steel. However, at high temperatures and high pressures (HTHPs), the corrosion of carbon steels in the presence of CO_2_ is a complex process. The formation of iron carbonate on the metal surface can significantly influence both the corrosion resistance of the metal and the adsorption of the inhibitor. Experiments are often designed to prevent or delay the formation of a protective iron carbonate layer, ensuring clear results. In these situations, detecting the inherent impact of an inhibitor can be challenging, and poor inhibition might go unnoticed due to misinterpretation of the reasons behind the decrease in corrosion rates. To accurately interpret inhibitor performance data, it is also important to consider the influence of inhibitors when iron carbonate partially or fully covers the steel surface. Therefore, a deeper understanding of this corrosion mechanism is essential in the oil and gas industry. Thus, this paper aims to demonstrate that XG can be used not only as a thickening agent in the EOR process but also as an active component in preventing sweet corrosion in the oil and gas industry.

To achieve this objective, thorough investigations into the anticorrosive properties of xanthan gum at high temperatures and pressures were conducted, employing weight loss and electrochemical measurement techniques across varying concentrations of xanthan gum and temperatures. Furthermore, the XG adsorption behavior on the metal surface, including the adsorption isotherm and activation parameters, was analyzed and discussed. Techniques such as Fourier-transform infrared spectroscopy (FTIR) and scanning electron microscopy (SEM–EDS) were used to characterize the metal surface and support the gravimetric and electrochemical results.

## 2. Experimental

### 2.1. Sample and Solution Preparation

This study was conducted on N80 carbon steel pipe with the following composition (wt.%): C 0.39%, Cr 0.04%, Cu 0.26%, Si 0.26%, Mn 1.80%, V 0.19%, Ni 0.04%, and Al 0.03%, with the remainder being Fe, and the microstructure is displayed in Figure S1. The pipe was machined into coupons measuring 20 mm × 10 mm × 2 mm, ground using various grades of silicon carbide paper, and polished to a surface finish of 1 µm. Subsequently, the specimens were washed ultrasonically with distilled water and absolute alcohol, then dried in an oven.

The tested solution consisted of 2 wt.% potassium chloride (KCl), prepared using analytical-grade KCl (Sigma-Aldrich, Darmstadt, Germany) and pure deionized water. According to ASTM Standard G111, the minimum volume-to-exposure area ratio (V/A) value for HTHP corrosion tests is 33 mL cm^−2^. Consequently, the autoclave was filled with 1000 mL of the test solution to ensure a V/A value not inferior to the recommended one. Four coupons were utilized in the experiment; three were used to measure the corrosion rate (CR) of the steel through weight loss, while one specimen was employed to observe the morphology of the corrosion product layers and analyze the phases of these layers. The exposed surface area of each coupon was 5.2 cm^2^. The specimens used for the electrochemical tests were machined into a cylindrical shape and embedded in PTFE, with an exposed surface area of 0.50 cm^2^. The gravimetric and electrochemical experiments were performed together to maintain a constant V/A ratio of 46.95 mL cm^−2^. CO_2_ was bubbled for 3 h to remove O_2_. After installing the specimens, the autoclave was closed, and CO_2_ was purged into the tested solution for 0.5 h. The temperature and pressure were then adjusted to the desired value. The purging was maintained throughout the test to minimize air ingress. All immersion and electrochemical tests were conducted with and without the addition of different concentrations of XG at temperatures of 30 and 90 °C, under a constant CO_2_ partial pressure of 5 bar. After saturation, the pH of the blank solution at 30 and 90 °C was 3.5 and 3.43, respectively. The pH values were measured with HP glass-based and HTHP ZrO_2_-based pH electrodes.

### 2.2. Weight Loss Measurements

The weight loss experiments were conducted under the test conditions mentioned above, by exposing the samples to the tested solution for 24 and 72 h. Before each test, the specimens were weighed using an analytical balance with an accuracy of 0.1 mg. After the immersion time had elapsed, the samples were extracted and rinsed with deionized water. Before weighing, the corrosion products were removed using a descaling solution composed of 500 mL of hydrochloric acid (sp gr 1.19), 500 mL of distilled water, and 3.5 g of hexamethylenetetramine [27]. The samples were ultrasonically washed with distilled water and absolute alcohol and then dried in the oven. The CR and inhibition efficiency percentage (IE%) were calculated using the following Equations (1) and (2) [6]:

(1) $$\mathrm{CR}\;(\mathrm{mm}\;y^{-1})\;=\frac{87.6\;∆m}{\mathrm{dAt}}$$

(2) $$\mathrm{IE}\;(\%)=\frac{\mathrm{CR}-CR^{\mathrm{inh}}}{\mathrm{CR}}\times 100$$

where Δ*m* represents the weight loss, *d* denotes the density of the metal, *A* refers to the surface area of the sample, and *t* indicates the immersion time. CR and CR^inh^ represent the corrosion rates of the steel without and with XG, respectively.

All experiments were conducted three times, and the average result was calculated.

### 2.3. Electrochemical Measurements

The electrochemical experiments were conducted under the test conditions mentioned above using a three-electrode system, featuring the N80 carbon steel sample as the working electrode (with an exposed surface area of 0.50 cm^2^) and the platinum foil as the counter electrode (CE). The reference electrode was an external pressure-balanced Ag/AgCl electrode with a 0.1 M KCl solution. All potentials are reported with respect to this reference electrode. The measurements were performed with a Gamry Reference 600 potentiostat. Impedance spectroscopy (EIS) was performed over a frequency range of 1 kHz to 10 mHz with an amplitude of 5 mV at open circuit potential (OCP) after different immersion times. Potentiodynamic polarization (PDP) measurements were conducted in a potential range of ±0.3 V vs. OCP with a scan rate of 1 mV s^−1^ after holding the specimen for 24 h with different concentrations of XG. The EIS data and the PDP parameters were analyzed using Echem Analyst 5.21 software (Gamry Instruments, Warminster, PA, USA). The IE% was calculated based on the polarization resistances (Rp) and corrosion current density (*i*_corr_) values using Equations (3) and (4) [6]:

(3) $$\mathrm{IE}\;(\%)=\frac{i_{\mathrm{corr}}-i_{\mathrm{corr}}^{\mathrm{inh}}}{i_{\mathrm{corr}}}\times 100$$

(4) $$\mathrm{IE}\;(\%)=\frac{R_{p}^{\mathrm{inh}}-R_{p}}{R_{p}^{\mathrm{inh}}}\times 100$$

where $R_{p}^{\mathrm{inh}}$, *R*_p_, $i_{\mathrm{corr}}^{\mathrm{inh}}$, and *i*_corr_ are the polarization resistance and corrosion current values in the presence and absence of the inhibitor, respectively.

### 2.4. Surface Analysis

The surface morphology of the samples was analyzed by immersing them in the aforementioned test conditions after 24 and 72 h, with or without 1.0 g L^−1^ XG. 

FTIR analysis was conducted using a Thermo Scientific Nicolet iS50 (Indianapolis, IN, USA) spectrophotometer equipped with an attenuated total reflectance (ATR) accessory. The FTIR spectra were recorded at a spectral resolution of 4 cm^−1^ with 256 co-added scans over the range from 4000 to 500 cm^−1^. SEM-EDS analysis was performed using a Tescan Mira (Tescan GmbH, Brno, Czech Republic) scanning electron microscope at varying magniﬁcations. Grazing incidence X-ray diffraction (GIXRD) was performed to determine the composition of the corrosion product scale. GIXRD was recorded with an incident angle of 3° and scanned in 0.02° steps over the range of 20° to 70° at room temperature.

## 3. Results and Discussion

### 3.1. Weight Loss Measurements

**Figure 1:**
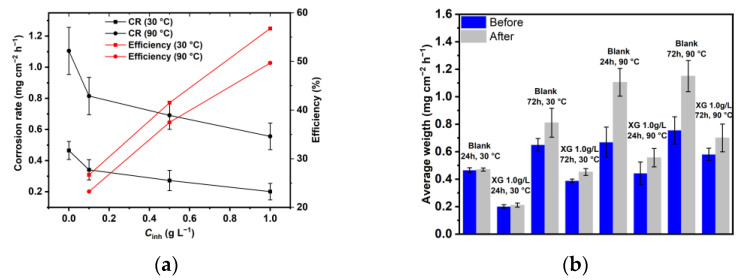
(**a**) Average corrosion rate and inhibition efficiency obtained from weight loss measurements at various concentrations of XG after 24 h of immersion at 30 and 90 °C. (**b**) Average corrosion rate at different immersion times and temperatures, before and after removing the corrosion products on the metal surface, in the presence of 1.0 g L^−1^ of XG.

The values of the average corrosion rate (ACR) calculated via weight loss measurement and the inhibition efficiency (IE%) observed with different concentrations of XG at various temperatures after immersing the samples under the tested conditions for 24 h are shown in Figure 1a and Table S1. The ACR was determined after carefully removing the corrosion product from the metal surface using a descaling solution [27]. The image clearly shows that XG can mitigate the corrosion of N80 carbon steel in a CO_2_-saturated saline solution, and its inhibitory action increases with concentration, reaching a maximum IE% value of 55.11% at a concentration of 1.0 g L^−1^. At 90 °C, XG continues to provide a reasonable degree of protection against sweet corrosion, although the protection efficiency decreases slightly, reaching a maximum IE% value of 49.68% at a concentration of 1.0 g L^−1^. This result is likely due to the weakened interactions between inhibitor molecules and the metal surface as the temperature increases.

A review of the current literature [28,29,30] reveals that the molecular conformation of XG chains in aqueous solution can exist in various forms, primarily as a rigid double-stranded helix and a flexible single-stranded coil conformation, depending on the ionic strength and/or temperature of the solution. In the helix structure, two single XG macromolecules are stabilized by hydrogen bonds. At high temperatures (e.g., above 80 °C), XG undergoes a denaturation transition from a rigid double-stranded helical to two single, more flexible, worm-like macromolecules (Figure S1). Consequently, the favorable IE observed at high temperatures may be attributed to the helix/coil transition process, which increases the number and mobility of XG macromolecules available for absorption on the surface, thereby partially compensating for the weakened interactions at the molecules/metal interface.

Figure 1b and Table S2 show the ACR calculated via weight loss measurement at different immersion times and temperatures before and after removing the corrosion products from the surface, in the presence of 1.0 g L^−1^ of XG. By analyzing weight loss data post-removal process, one can observe the impact of XG at various immersion durations and temperatures. As depicted in the figure, at 30 °C, the corrosion rate increases as the immersion time increases. The uninhibited sample exhibits significant weight loss after prolonged immersion, with a large change in mass loss following the removal of the corrosion products. This variation in mass signifies the development of a loose and non-protective corrosion product layer on the surface, as confirmed by the cross-sectional analysis presented in the surface analysis section. As reported in the literature, low temperatures (e.g., 30–50 °C) restrict the formation of a protective corrosion scale; as a result, corrosion intensifies as immersion time increases [31,32,33].

Increasing the temperature, the data clearly show that the corrosion rate of the uninhibited sample rises, compared to the one observed at 30 °C. However, at high temperatures (e.g., above 80 °C), the precipitation of FeCO_3_ becomes thermodynamically more favorable, even during short immersion times. At 90 °C, and after 24 h of immersion, a significant weight variation can be observed after removing this layer. After 72 h, the corrosion rate is relatively stable, and the weight variation is similar to that observed at 24 h, indicating that the corrosion product layer formed protects the metal surface from further corrosion. The considerable weight variation observed at both immersion times can be attributed to the formation of an adherent and thick layer of corrosion products. At an increased temperature, and during the early stages of the experiment, when corrosion products have not yet formed, the kinetics of all involved corrosion processes are accelerated [31]. However, as the corrosion continues, a protective layer starts to form on the metal surface, which slows the corrosion rate of the metal, in agreement with the EIS performed at different immersion times reported in Section 3.4.

In contrast, in the presence of XG, the figure shows a slight difference in mass loss before and after the removal of the corrosion products, regardless of the time and temperature. This observation indicates that the adsorption of XG significantly hinders the corrosion processes on the metal surface from the early stage, consequently reducing the growth of the corrosion products.

Table S2 presents the IE% of various natural polysaccharides used as corrosion inhibitors across different media. It is important to note that most reported studies were conducted over short immersion times (i.e., up to 6 or 12 h), with only a few extending to longer durations (i.e., 24 h). The table indicates that, compared to other natural corrosion inhibitors, XG can be regarded as an effective and environmentally friendly corrosion inhibitor for carbon steel in a CO_2_-saturated chloride solution, even after extended exposure times.

### 3.2. Adsorption Study and Activation Parameters

**Figure 2:**
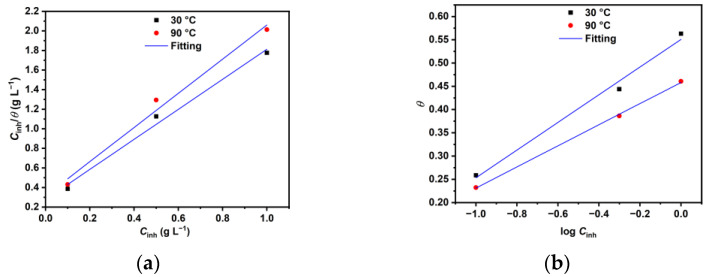
Adsorption isotherms: (**a**) Langmuir and (**b**) Temkin.

To gain a deeper understanding of the adsorption mechanism, various adsorption models were employed to fit the results of the weight loss experiments. The best fit was obtained with the Langmuir (Equation (5)) [8,16,18] and Temkin (Equation (6)) [11,34] models, as shown in Figure 2.

(5) $$\frac{C_{\mathrm{inh}}}{\theta }=\frac{1}{K_{\mathrm{ads}}}+C_{\mathrm{inh}}$$

(6) $$\exp\;(-2a\theta )\;=K_{\mathrm{ads}}C_{\mathrm{inh}}$$

where *K*_ads_ is the constant related to adsorption energy, *C*_inh_ is the concentration of the inhibitor, *a* is the adsorbate interaction factor, and *θ* is the surface coverage [16,18]. *K*_ads_ calculated from the adsorption isotherms can be linked to the free energy of adsorption (${∆G}_{\mathrm{ads}}^{{}^{\circ}}$) by the following relationship [8,26]:

(7) $${∆G}_{\mathrm{ads}}^{{}^{\circ}}=-\mathrm{RT}\ln(10^{3}\;K_{\mathrm{ads}})$$

where the value 1 × 10^3^ is the concentration of water molecules in solution expressed in g L^−1^.

The adsorption isotherm parameters data are listed in Table 1, which shows that the Langmuir isotherm yields higher values of the linear correlation coefficient (*R*^2^) for the tested temperatures, indicating that this isotherm is the most suitable for explaining how XG is adsorbed onto the metal surface. Similar results were also reported in other studies involving the use of XG as a corrosion inhibitor in various solutions [16,18,26]. However, it should be noted that the selection of a given isotherm should not be based solely on the value of *R*^2^ but rather on the assumptions that led to its development. For example, the Langmuir isotherm model was developed under the assumption that (I) there is no interaction between the adsorbed molecules and (II) there is a fixed number of adsorption sites on the metal surface, and each of them holds one adsorbed species [35,36]. The value of *R*^2^ diverged from unity, which likely indicates the interaction of adsorbed XG molecules on the N80 steel surface [12]. For these reasons, the Temkin adsorbed isotherm, which accounts for the omitted interaction parameter by the Langmuir isotherm, was used to fit the WL measurement [12,36]. It follows from Table 1 that all values of ${∆G}_{\mathrm{ads}}^{{}^{\circ}}$ are negative, inferring that the adsorption of XG is a spontaneous process and that the adsorption mechanism involves a combination of physical and chemical adsorption, with physical adsorption being the more prevalent type. Additionally, the values of the adsorbate interaction factor *a* are negative at both temperatures, indicating repulsive forces between the adsorbed macromolecules [6,34]. The findings are in agreement with previous studies [16,17,18,26].

The dependence of the corrosion rate on temperature can be expressed using the condensed Arrhenius Equation [5,6]:

(8) $$\log\frac{{\mathrm{CR}}_{2}}{{\mathrm{CR}}_{1}}=\frac{E_{a}}{2.303\;R}\left(\frac{1}{T_{1}}-\frac{1}{T_{2}}\right)$$

where CR_1_ and CR_2_ represent the corrosion rates at temperatures *T*_1_ (30 °C) and *T*_2_ (90 °C), respectively. *E*_a_ denotes the apparent activation energy. The heat of adsorption values were calculated using the following equation [5,6]:

(9) $$Q_{\mathrm{ads}}=2.303\;R\left[\log\left(\frac{{\;\theta }_{2}}{1-\theta_{2}}\right)-\log\left(\frac{\theta_{1}}{1-\theta_{1}}\right)\right]\times \left(\frac{T_{1}\times T_{2}\;}{T_{2}-T_{1}}\right)$$

where *θ*_1_ and *θ*_2_ represent the values of the degree of surface coverage at the tested temperatures. The calculated parameters are listed in Table 2.

The literature has reported that, for higher *E*_a_ values in the presence of the inhibitor, the adoption process is regarded as a physical adsorption process. In comparison, lower or equal *E*_a_ values support the chemisorption adsorption process [6,8,26]. The table shows that, in the presence of XG, the values of *E*_a_ are higher than those in the blank solution, likely due to the XG adsorptive layer, which reduces the exposed surface area available for corrosion [6,8,26]. Furthermore, these values increase with the concentration of XG, which may indicate that the activation energy primarily controls the inhibition process [18,26]. It is essential to note that, although the values of *E*_a_ in the presence of XG are higher than those observed for the blank solution, the difference is minimal. These findings, along with the values of ${∆G}_{\mathrm{ads}}^{{}^{\circ}}$, suggest that the adsorption process of XG can be described as a mixed-type adsorption (i.e., physical and chemical adsorption); however, physical adsorption appears to be the dominant mechanism.

### 3.3. Electrochemical Measurements

**Figure 3:**
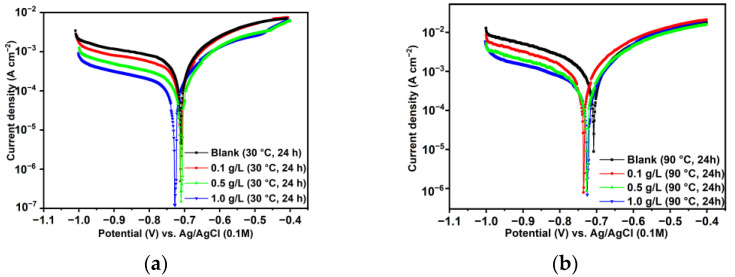
Potentiodynamic polarization curves in the absence and presence of different concentrations of XG after 24 h of immersion time at (**a**) 30 °C and (**b**) 90 °C.

The potentiodynamic polarization experiments conducted after exposing the sample to the tested condition for 24 h, with and without the presence of XG, are shown in Figure 3, with the kinetic parameters listed in Table 3. The images and data show that, at lower temperatures (Figure 3a), the addition of XG to the system affects the corrosion current density (*i*_corr_), with its value decreasing as the XG concentration increases, reflecting a maximum corrosion inhibition efficiency of 73.63% at 1.0 g L^−1^. The IE% values at different concentrations of XG were calculated using Equation (3). It is worth mentioning that the anodic branches do not exhibit a well-defined Tafel region; therefore, the values of *i*_corr_ were estimated by extrapolating the cathodic Tafel region, in agreement with Amin et al. [37].

In an aqueous solution, CO_2_ gas forms carbonic acid [38]:

(10) $$CO_{2}+H_{2}O_{\left(l\right)}↔H_{2}CO_{3}$$

This, in turn, dissociates into bicarbonate and then carbonate anions:

(11) $$H_{2}CO_{3}↔{HCO_{3}^{-}+H}^{+}$$

(12) $$HCO_{3}^{-}↔CO_{3}^{2-}+H^{+}$$

lowering the pH of the solution, and hence, leading to the following cathodic reactions [38]:

(13) $${2H}^{+}+2e^{-}\to H_{2}$$

(14) $$2H_{2}CO_{3}+2e^{-}\to {2HCO_{3}^{-}+H}_{2}$$

(15) $$2HCO_{3}^{-}+2e^{-}\to 2CO_{3}^{2-}+H_{2}$$

(16) $$2H_{2}O+2e^{-}\to 2OH^{-}+H_{2}$$

In a CO_2_-saturated solution, the anodic reaction of the steel is a multi-step process (a consecutive mechanism) [38]:

(17) $$\mathrm{Fe}+H_{2}O\to {\mathrm{FeOH}}_{\left(\mathrm{ads}\right)}+{H^{+}+e}^{-}$$

(18) $${\mathrm{FeOH}}_{\left(\mathrm{ads}\right)}\to {\mathrm{FeOH}}^{+}+e^{-}$$

(19) $${\mathrm{FeOH}}^{+}+H^{+}\to {\mathrm{Fe}}^{2+}+H_{2}O$$

At lower pH (e.g., <4), Equation (13) is the primary reaction due to the high concentration of H^+^. When the pH ranges from 4 to 6, Equations (14) and (15) become the principal cathodic reactions. Finally, at a pH higher than 9, the dominant cathodic reaction is a reduction in water, represented by Equation (16). Bockris et al. [39] proposed a consecutive mechanism for the dissolution of iron in the presence of CO_2_, with the rate-determining step being described by Equations (17)–(19).

Figure 3a shows an active dissolution feature in the anodic region of the polarization curves. Simultaneously, the cathodic region of the polarization curves exhibits a diffusion process-like current shoulder [40]. The literature attributes this phenomenon to the non-homogeneous reaction of CO_2_ hydration on the metal surface in a CO_2_-saturated environment [5,40]. With the addition of XG, the cathodic polarization current shoulder decreased, while the anodic current density also showed a reduction. In this study, the initial pH value of the solution was determined to be 3.5; hence, the decrease in both the cathodic and anodic branches in the presence of XG implies that both cathodic reduction (Equations (13) and (14)) and the anodic dissolution (Equations (17)–(19)) reactions were inhibited by XG [38]. This result can likely be ascribed to the adsorption of XG macromolecules at the metal/solution interface, which slowed down both cathodic and anodic reactions by blocking the respective active sites on the metal surface, consequently affecting the metal’s dissolution [13,34].

At 90 °C, the PDP plots exhibit the same characteristics observed at 30 °C, with the values of *i*_corr_ decreasing as the XG concentration increases (Figure 3b). Notably, XG had a greater impact on the cathodic reaction, which may indicate that XG was more likely to adsorb on the cathode sites, thereby preventing the hydrogen evolution reaction, as discussed in more detail in Section 3.6. IE% was found to decrease slightly with an increase in temperature at all concentrations, supporting the proposed physisorption/chemisorption adsorption mechanism. At both temperatures, the corrosion potential (*E*_corr_) values with the addition of XG shifted to more negative values compared to the free-inhibitor solution, although the magnitude of this shift is negligible (e.g., <0.085 V [8]). This result indicates that XG functions as a mixed-type inhibitor, in agreement with previously published findings [16,18,26]. The PDP results are consistent with those observed from the gravimetric measurements.

**Figure 4:**
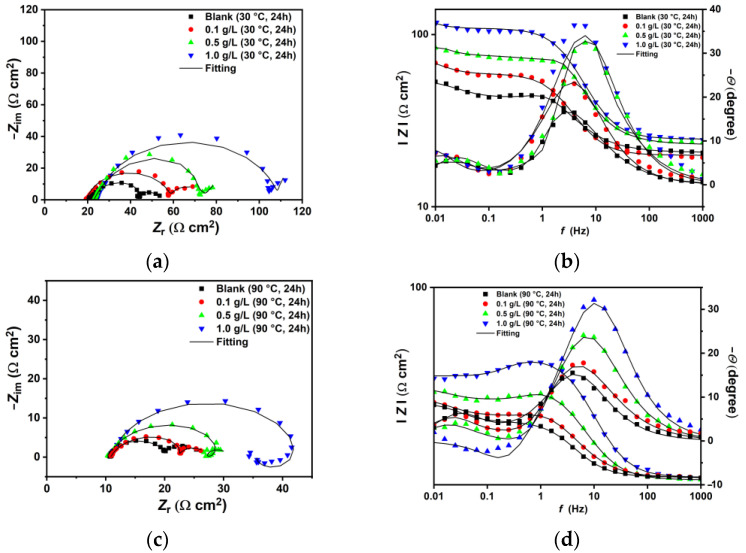
EIS plots in the absence and presence of different concentrations of XG after 24 h of immersion time at: (**a**,**b**) 30 °C and (**c**,**d**) 90 °C.

**Figure 5:**
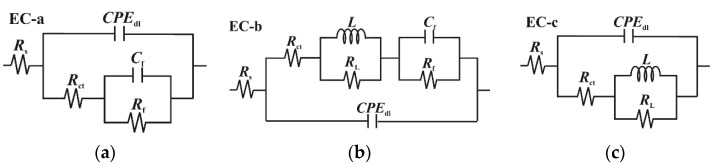
Equivalent circuits used to fit experimental data. (**a**) T = 30 °C, and at 90 °C for the blank and lower concentrations of XG (e.g., up to 0.1 g L^−1^); (**b**) at 90 °C, with 0.5 g L^−1^ of XG; (**c**) at 90 °C, with 1.0 g L^−1^ of XG.

The EIS plots conducted at 30 °C and 90 °C are shown in Figure 4. At 30 °C, the blank solution displays two time constants: one from high (HF) to medium frequencies (MF) and the second at low frequencies (LF) (Figure 4a). The capacitive loop at HF-MF has been linked to electron transfer and the charge–discharge processes of the electrical double layer between the electrode surface and the electrolyte solution [34]. The second loop at LF corresponds to the capacitance and resistance of corrosion products. The data were fitted using the equivalent circuits (EC) shown in Figure 5, in accordance with observations from similar studies in the literature [2,3,9,27,40,41,42]. The EIS spectra at low temperature were fitted with the EC-a model, where *R*_s_ represents solution resistance, and *CPE*_dl_ and *R*_ct_ refer to the constant phase element of the double-charge layer capacitance and the charge transfer resistance, respectively. *C*_f_ and *R*_f_ denote the capacitance and resistance of the corrosion products and/or adsorbed inhibitor film [43]. The EIS-fitting data and the chi-squared (*χ*^2^) values are listed in Table 4. The Nyquist plot shows that the HF-MF capacitive loop steadily increases after the addition of XG under the tested conditions, with the *R*_ct_ value rising from 24.19 Ω cm^2^ to 89.38 Ω cm^2^ for the blank and 1.0 g L^−1^, respectively. The Bode plots (Figure 4b) indicate that the free-inhibitor solution displays a small, narrow single peak in the phase angle plot, with a maximum at approximately 17°. By contrast, in the presence of XG, the phase angle becomes increasingly wider, and its maximum value rises to approximately 38°. In this study, the *CPE* elements served as an alternative to capacitance elements (*C*) to account for the inhomogeneity of the corroded surface. The impedance of a *CPE* element comprises two parameters, namely *Y*_o_ and *n* (Equation (S1)). *Y*_o_ measures capacitance, while *n* indicates the degree of deviation from ideal capacitive behavior, which also reflects the smoothness of the surface [3]. Based on the model proposed by Brug et al. [44], *CPE*_dl_ can be used to calculate the double-layer capacitance (*C*_dl_) (Equation (S2)). The data listed in Table 4 indicate that *C*_dl_ decreases as the concentration of XG increases. Similarly, the *n*_dl_ parameter was influenced by the addition of XG, which exhibits higher values in its presence compared to the free-inhibitor solution [3,8], inferring a smoother and less damaged surface compared to the blank solution, as corroborated by the SEM analysis. Moreover, the *R*_f_ values of the inhibited solutions were greater than those of the blank, suggesting the development of a less conductive film with minimal porosity that retains the adsorbed inhibitor molecules, unlike the highly porous and conductive thin film observed in the blank solution [45]. Various studies have reported similar findings regarding XG as a corrosion inhibitor, attributing this phenomenon to the adsorption of XG molecules on the metal surface [8,16,17,18,22,24,25,26]. The authors noted that the adsorptive inhibitor film alters the dielectric properties of the steel/solution interface according to the Helmholtz model (Equation (S3)), which reduces the rates of both cathodic and anodic reactions [3,6]. The IE% values at different concentrations of XG were calculated based on the polarization resistance values (*R*_p_ = *R*_ct_ + *R*_f_) using Equation (4). The findings indicate that XG enhances the corrosion resistance of N80 under the tested conditions, achieving a maximum efficiency of 70.10% at a concentration of 1.0 g L^−1^ of XG.

At 90 °C, the EIS spectra exhibit a similar trend observed at lower temperatures, characterized by a gradual increase in the capacitive loop associated with the charge transfer resistance as the XG concentration increases (Figure 4c,d). However, the plots show different behavior at various XG concentrations. For instance, the EIS spectra for the blank and at 0.1 g L^−1^ of XG are similar to those observed at lower temperatures, featuring two capacitive loops related to *R*_ct_ and *R*_f,_ and are therefore fitted with the EC-a model. However, an increase in the XG concentration (e.g., 0.5 g L^−1^) resulted in the emergence of an inductive loop at MF, followed by a capacitive loop at LF, which was fitted with the EC-b model. For a further increase in the XG concentration (e.g., 1.0 g L^−1^), the inductive loop was shifted to LF, and the Nyquist plot is characterized by a broad depressed semicircle at HF-MF, followed by an inductive loop to LF (equivalent circuit fitting EC-c model). The inductive loop is related to the adsorption of intermediate species on the metal surface. This adsorption occurs as a result of the anodic dissolution of steel (e.g., FeOH_(ads)_, Equations (17)–(19)) [2,40,41,42,46], and/or, in the presence of inhibitors, due to the relaxation process caused by the adsorption of inhibitor molecules on the electrode surface [47,48]. Belarbi et al. [46] suggested that the observed shift can be explained by the processes of adsorption and desorption of these intermediate species on the metal surface, which affect the rates of the cathodic and anodic reactions. Under the testing conditions, the sample exposed to the free-inhibitor and low-inhibitor concentration solution corrodes quickly, and its surface is rapidly covered with a layer of corrosion products. This layer occupies the adsorption sites for FeOH_(ads)_, resulting in the absence of any inductive loop. At 0.5 g L^−1^ of XG, the *R*_ct_ value for the inhibited solution is higher than that of the blank solution, indicating that the inhibitor slows down the corrosion processes occurring at the metal/solution interface. Therefore, the presence of the inductive loop at the MF range may be attributed to a competitive adsorption process between FeOH_(ads)_ and the XG molecules. Furthermore, as the concentration of XG increases, the *R*_ct_ value continues to rise to the entire frequency range, indicating that XG effectively covers most of the metal surface and further inhibits the corrosion process. These findings are in agreement with the other results reported in this manuscript, indicating that the thickening agent XG continues to provide adequate protection against sweet corrosion even at 90 °C.

### 3.4. Effect of Time

**Figure 6:**
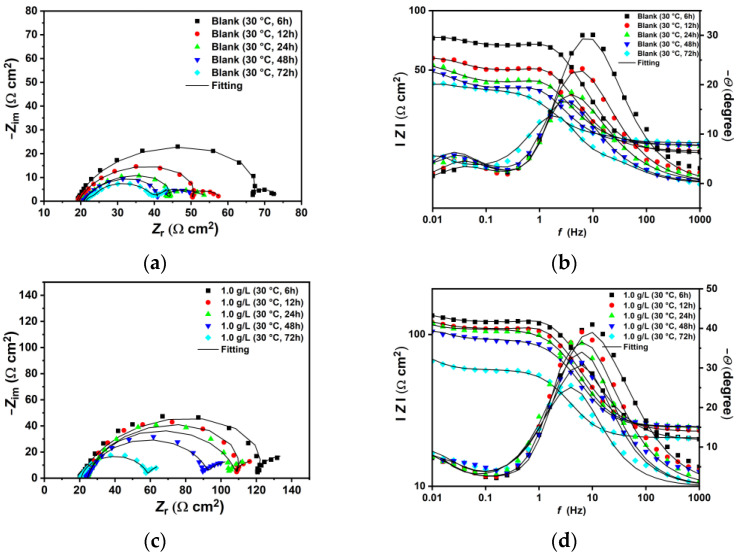
EIS plots carried out without (**a**,**b**) and with the presence of 1.0 g L^−1^ of XG (**c**,**d**) at 30 °C at different immersion times.

**Figure 7:**
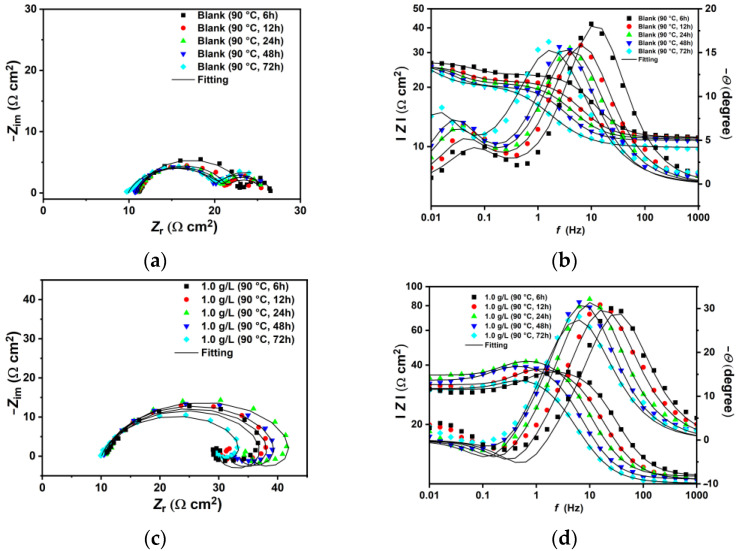
EIS plots carried out without (**a**,**b**) and with the presence of 1.0 g L^−1^ of XG (**c**,**d**) at 90 °C at different immersion times.

Figure 6 and Figure 7 show the EIS measurements carried out at different immersion times under the tested conditions, both without and with 1.0 g L^−1^ of XG, at 30 and 90 °C. The EIS plots have been fitted with ECs, as shown in Figure 5, and the obtained parameters are presented in Tables S3 and S4. The data indicate that, at 30 °C, in both uninhibited and inhibited solutions, the *R*_p_ value decreases over time. During the initial corrosion of carbon steel, after the ferrite phase dissolves, the Fe_3_C phase remains, and as corrosion progresses, it accumulates on the steel surface. This phase will act as a cathodic phase, accelerating steel corrosion over time [6,8]. As a result, the *R*_p_ values decrease during the early stage. However, compared with the blank solution, it is evident that XG demonstrates its effectiveness within the first few hours of the experiment (i.e., 6 h) with an IE% of around 56%. This indicates rapid adsorption of XG, providing quick protection for the metal surface. IE% increases steadily, reaching a peak of 70% after 24 h, before gradually declining to 52.45% after 72 h.

At 90 °C, for the uninhibited solution, *R*_p_ values remain constant over time. This indicates that, after an initial corrosion stage—just after the first 6 h—the metal surface is covered by a layer of corrosion products that can slow down the corrosive processes on the surface. In contrast, when XG is present, *R*_p_ values consistently increase, with IE% reaching a maximum value of 61.41% after 24 h, then decreasing to 40.71% after 72 h of immersion. Table S2 shows that, compared to other natural corrosion inhibitors, XG is a good, eco-friendly option for preventing the corrosion of carbon steel against sweet corrosion over extended periods.

### 3.5. Surface Analysis

The surface of the N80 carbon steel sample was characterized after 24 and 72 h of exposure under the tested conditions, both with and without 1.0 g L^−1^ of XG. Surface analysis was conducted using scanning electron microscopy (SEM) and X-ray diffraction (XRD).

**Figure 8:**
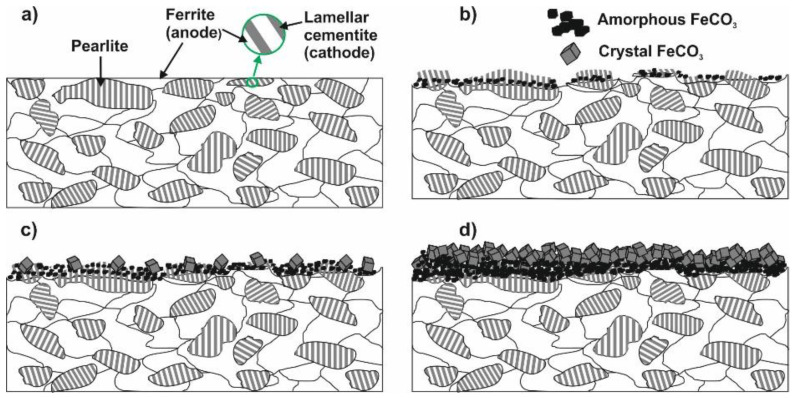
Schematic diagrams showing the formation of corrosion scales on carbon steel N80 immersed in CO_2_-saturated saline solution as a function of time and temperature. (**a**) Polished metal; (**b**) revealing of the cementite matrix; (**c**) nucleation of FeCO_3_; (**d**) formation of FeCO_3_.

**Figure 9:**
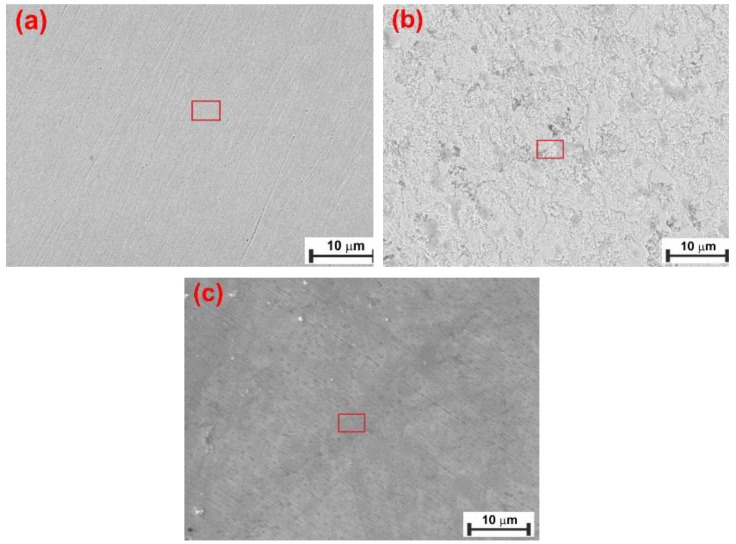
SEM and EDS (red square) analysis of the tested steel surface: (**a**) polished sample, and after 24 h of immersion (**b**) without and (**c**) with 1.0 g L^−1^ of XG solution at 30 °C.

**Figure 10:**
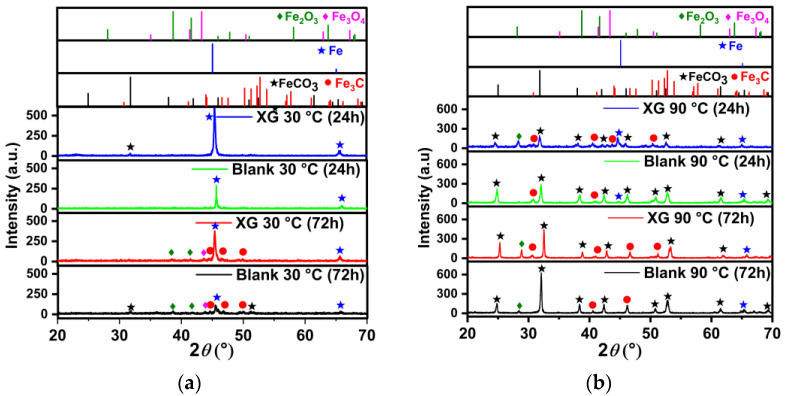
XRD spectrum of corrosion products of N80 steel at different immersion times: (**a**) 30 ℃ and (**b**) 90 ℃. (FeCO_3_, ICDD:29-0696; Fe_3_C, ICDD:35-0772; Fe, ICDD:06-0696).

The scheme of the corrosion process depicted in Figure 8 can be used to understand the corrosion phenomenon occurring on the N80 surface. N80 is a ferrite-perlite steel, with the letter accounting for approximately 41% of the total microstructure (Figure S2). The perlite phase, in turn, consists of a lamellar structure of cementite (Fe_3_C) embedded in a ferrite matrix (Figure 8a). The gravimetric experiments demonstrated that the steel corroded under the tested conditions, with an average corrosion rate of 0.470 mg cm^−2^ h^−1^ determined after 24 h of immersion. Corrosion occurred due to the potential difference between the cementite structure (i.e., the nobler phase) and the adjacent ferrite matrix, resulting in the dissolution of the latter phase and leaving undissolved Fe_3_C exposed to the metal surface (Figure 8b), as confirmed by the SEM analysis presented in Figure 9b compared to the freshly polished surface (Figure 9a). The EDS analysis presented in Table S5 indicates that the metal surface was covered by a layer of corrosion product primarily composed of carbon (6.0 wt.%), iron (89.3 wt.%), and other alloying elements of the steel, with only a small amount of oxygen (1.5 wt.%). These findings suggest the presence of Fe_3_C on the sample surface; FeCO_3_ was not observed in the corrosion products, which is consistent with the GIXRD analysis (Figure 10a) and with the literature [6,8,27,49]. In contrast to the blank solution, the adsorption of XG causes the metal surface to become significantly smoother, with abrasive scratches still visible (Figure 9c). 

**Figure 11:**
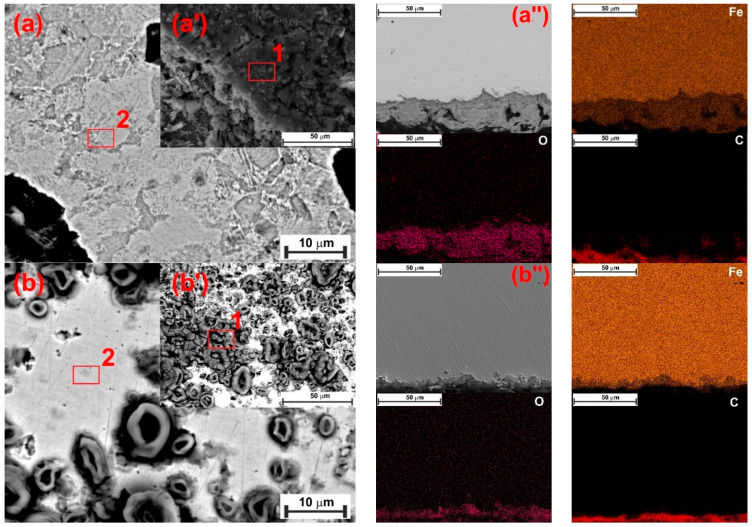
SEM and EDS (red square) analysis of the tested steel surface after 72 h of immersion without (**a**) and with 1.0 g L^−1^ of XG (**b**) at 30 °C.

After 72 h of immersion, the CR of the specimen exposed to the blank solution increased to 0.811 mg cm^−2^ h^−1^. As the immersion time increased, Fe_3_C accumulated on the metal surface, thereby increasing the cathode area, which, in turn, further accelerated the dissolution of the steel [6,49]. The SEM analysis after 72 h for the blank solution is displayed in Figure 11. It can be seen from the figure that the surface was covered by a thick corrosion product layer (Figure 11a’). Upon removing the corrosion product layer, the undissolved Fe_3_C protruding Fe_3_C structure is clearly visible (Figure 11a). At the same immersion time, the specimen exposed to the inhibited solution still exhibited a smoother surface; however, signs of damage began to appear on its surface (Figure 11b). The level of corrosion that occurred can be assessed by measuring the thickness of the corrosion layer. The cross-sectional analysis revealed that, in the presence of XG (Figure 11b’’), there was a thinner and more uniform corrosion product layer (e.g., ~4 µm) compared to the blank solution (e.g., ~22 µm) (Figure 11a’’). EDS analysis shows that the Fe content is 59.2 wt.% for the blank and 70.9 wt.% for the XG solution (Table S5). The rise in steel dissolution suggests a greater loss of Fe atoms from the steel surface as a result of the attack of Cl^-^ ions. This may explain the observed decrease in Fe content [9]. These results align with the GIXRD patterns, which show that the intensity of the characteristic diffraction peaks at 2θ = 45.05° and 65.93° for Fe decreased significantly in the uninhibited solution due to the masking effect of the corrosion product layer. Conversely, in the presence of the XG, the intensity of the Fe matrix peaks slightly decreased, due to a thin corrosion product layer that allows X-rays to penetrate the corrosion product film and generate the diffraction matrix [7]. These findings strongly suggest that the XG adsorption can mitigate the corrosion of the metal, even during prolonged immersion times, significantly influencing the formation of the corrosion product layer. Additionally, GIXRD analysis reveals the presence of small traces of FeCO_3_. Previous studies have shown that FeCO_3_ precipitates primarily at lower temperatures in environments with high pH (e.g., above 6) and/or elevated Fe^2+^ ion concentrations, as well as for long immersion times (e.g., above 168 h) [29]. Dugstad [50] observed that increasing the solution’s pH drastically lowers the Fe^2+^ ion concentration needed to surpass the FeCO_3_ solubility product, thus helping its precipitation. In this study, pH rose from 3.5 at the start to 5.2 after 72 h of immersion, making FeCO_3_ precipitation more likely. However, the author noted that the Fe^2+^ ion concentration is higher at the surface/solution interface (Figure 8b, between the protruding lamellae) than in the bulk solution, indicating that less Fe^2+^ is required to promote FeCO_3_ formation on the metal surface, thereby increasing the chance of FeCO_3_ presence, even at low pH.

**Figure 12:**
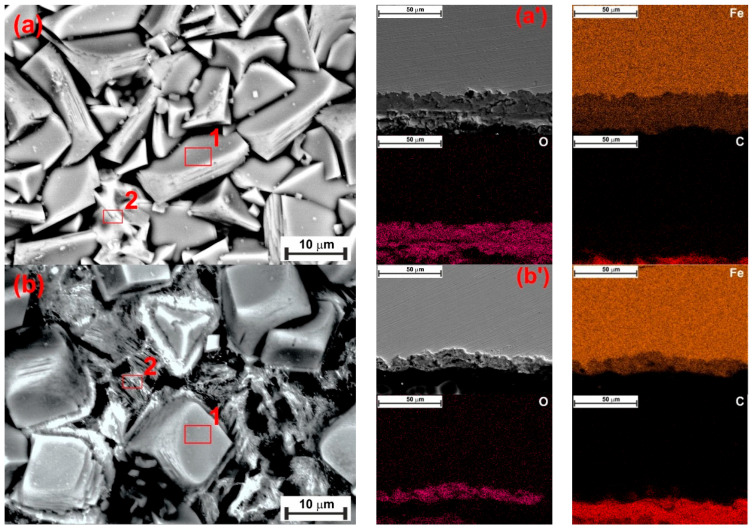
SEM and EDS (red square) analysis of the tested steel surface after 24 h of immersion without (**a**) and with 1.0 g L^−1^ of XG (**b**) at 90 °C.

Another factor that influences the precipitation of FeCO_3_ is the temperature. It has been reported that the precipitation of FeCO_3_ is thermodynamically more favorable with rising temperatures, even at low immersion times (Figure 8c,d) [31,32,33]. Top-view SEM analysis (Figure 12a) revealed the presence of FeCO_3_ crystals covering the entire steel surface for the sample exposed for 24 h to the blank solution at 90 °C. In contrast, sparse FeCO_3_ crystals formed on a Fe_3_C layer when XG was added (Figure 12b). The findings agree with the XRD patterns (Figure 10b), which indicate that the only identifiable crystalline compounds were FeCO_3_ and Fe_3_C. The presence of XG reduced the growth of FeCO_3_ crystals, suggesting that XG significantly hinders the corrosion. Similar results have also been reported in the literature [2,10,51,52]. The authors indicated that the adsorbed inhibitor slowed down the corrosion rate, thereby preventing the continuous supply of sufficient Fe^2+^ ions necessary for the growth of the FeCO_3_ film (Figure 8c). Consequently, the development of the FeCO_3_ film was significantly hindered. This is also evident in the formation of a thinner corrosion product layer (~11 µm), compared to the uninhibited solution (~30 µm), as observed via cross-sectional analysis (Figure 12a’,b’). These results confirmed the gravimetric and electrochemical observations, which have shown an inhibition efficiency of ~60% after 24 h of immersion at 90 °C. The high IE% value observed in the presence of XG can be attributed to its inhibiting effects rather than the protective effect of the corrosion product film.

**Figure 13:**
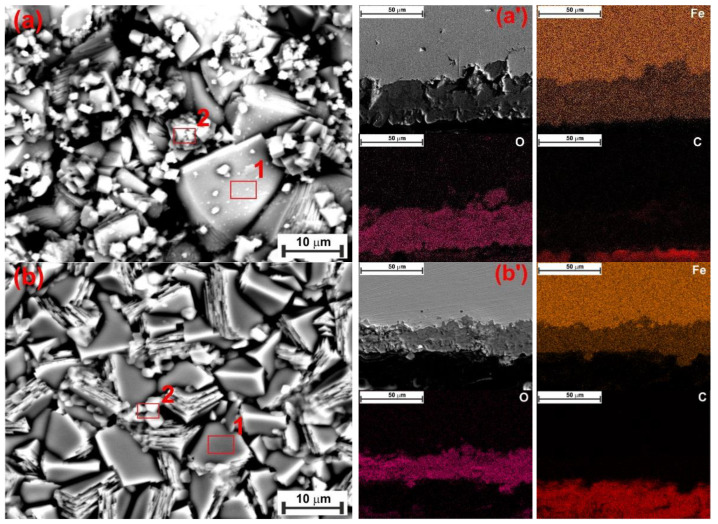
SEM and EDS (red square) analysis of the tested steel surface after 72 h of immersion without (**a**) and with 1.0 g L^−1^ of XG (**b**) at 90 °C.

After 72 h, in the presence of XG, nevertheless, the metal corrodes (Figure 13b), and the specimen surface appears to be covered by a more compact FeCO_3_ crystal layer, whereas holes and gaps can still be seen on the specimen surface exposed to the blank solution at the same immersion time (Figure 13a). These gaps can easily permit the diffusion of corrosive substances to the metal surface, resulting in a significant tendency for corrosion to occur. This phenomenon is evident in the corresponding cross-sectional images, which show that without XG, the interface between the corrosion product and bare steel is rougher, possibly due to the accumulation of several localized attacks (Figure 13a’,b’) [31,41,53,54]. Moreover, the cross-sectional images reveal a thick, double-layered corrosion product structure, consisting of an outer and inner layer. The literature indicates that the inner layer is likely composed of mixed phases, with FeCO_3_ embedded into the lamellar Fe_3_C matrix and the outer layer of FeCO_3_ [41,53,54]. Notably, when XG is present, the corrosion product thickness decreases to approximately 24 µm, compared to about 45 µm in the solution without XG. SEM analyses corroborate the electrochemical observations, showing that XG remains capable of mitigating CO_2_ at higher temperatures even after prolonged immersion times.

### 3.6. Mechanism of Inhibition

**Figure 14:**
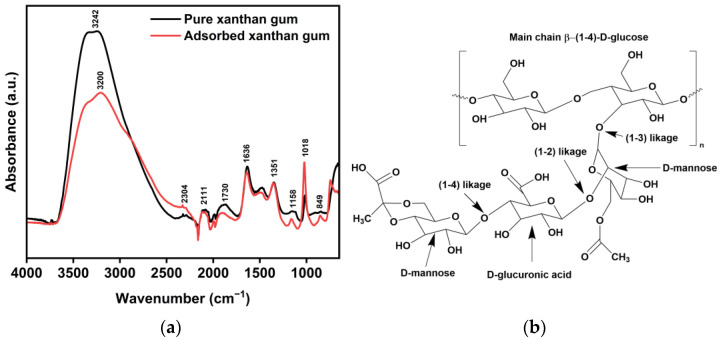
(**a**) FT-IR spectra of the native XG and surface-adsorbed XG. (**b**) Chemical structure of XG.

FTIR spectroscopy was employed to obtain crucial information about the interaction modes between the functional groups of the XG macromolecules and the metal surface. The stacked FTIR spectra of XG and the adsorbed XG on the metal surface after 24 h of immersion are illustrated in Figure 14a. The chemical structure of XG is displayed in Figure 14b. For the spectrum of pure XG, at 3242 cm^−1^, there is a broad peak attributed to intramolecular hydrogen bonds and the stretching vibration of O−H in the sugar ring of the XG molecule. At 2304 cm^−1^, a stretching vibration peak is observed for the C−H group. The region from 1800 to 800 cm^−1^ is recognized as the standard pattern range for polysaccharides. In brief, at 1730 cm^−1^, we see the −C=O stretching vibration associated with alkyl lipid; at 1636 cm^−1^, there is the asymmetric vibration of –COO–; at 1351 cm^−1^, there is the bending vibrational peak of –CH_2_–; and at 1158 and 1018 cm^−1^, we can observe the stretching vibration of –CH_2_–O–CH_2_– on the glycosidic linkage. These results align with the reported data [16,17,19,21,55,56]. The spectrum of the adsorptive layer of XG on the metal surface exhibits comparable characteristics to the native XG spectrum. However, the relative intensity of the peaks has changed, and there is a shift within the same frequency range. This suggests possible adsorption of XG on the metal surface [3]. The findings are consistent with earlier studies regarding the adsorption of compounds on steel surfaces [3,6,8,9,17,47].

**Figure 15:**
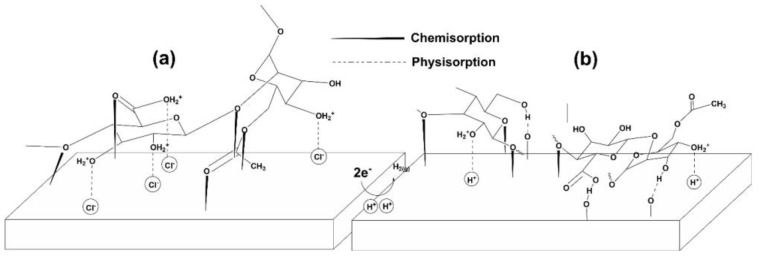
Schematic representation of the possible adsorption mechanism of XG on N80 steel: (**a**) physisorption and chemisorption and (**b**) H-bond formation.

The schematic model shown in Figure 15 can help explain the potential adsorption mechanisms occurring on the metal surface. The findings in this study indicate that XG can mitigate sweet corrosion at both low and high temperatures, with the adsorption process involving a combination of chemical and physical mechanisms, where the physical mechanism is more dominant. Based on the experimental findings and a review of the current literature on the adsorption process of polysaccharide-like inhibitors, it is shown that the formation of the adsorption film mainly occurs through the following mechanisms [6,8,16,17,26,34]: (I) electrostatic metal–inhibitor interaction; (II) unshared electrode pair interaction; and (III) a combination of all three.

(I) As seen in the FT-IR analysis, the peak intensity associated with inter/intramolecular H-bonded –OH (i.e., 3242 cm^−1^) decreases after adsorption of XG on the metal surface. The decrease in the intensity of this specific peak may result from various factors:
  - In acidic environments, the numerous –OH groups in XG molecules can be readily protonated. According to the literature [6,8,13,34], the drop in intensity may be due to electrostatic interactions between some of the protonated –OH groups of XG and the metal surface, with chloride ions adsorbed on the positively charged metal acting as bridges between the two.
  - The change can also be attributed to the formation of H-bonds between the XG’s hydroxyl groups and H^+^ absorbed on the cathodic sites of the steel surface through hydrogen bonding, as illustrated in Figure 15b. Consequently, H_2_ evolution is reduced (Equation (14)), as confirmed by the drop in cathodic current density in the presence of XG [6,8,13,34].
  - The inhibitor may also interact with the FeCO_3_ layer formed on the metal surface. Shaikhah et al. [57] suggested, using density functional theory analysis, that the inhibitor could adsorb to the FeCO_3_ layer through hydrogen bonding. These bonds occur between the hydroxyl and carboxylic groups and the oxygen atoms on the FeCO_3_ layer (Figure 15b).

Moreover, XG is an anionic polysaccharide because the pyruvic and glucuronic units have negatively charged carboxyl groups [20,29]. The presence of negatively charged side chains encourages the attachment of XG to the metal surface through electrostatic interaction forces.

(II) XG contained numerous electronegative heteroatoms (O) and oxygen functional groups (e.g., –OH, –COOH, –CH_2_–O–, and –CH_2_–O–CH_2_–), and unsaturated bonds (–C=O), with unshared electrons. The FT-IR spectra of the surface-adsorbed XG show some changes. The initial physisorption process allows XG to attach to the surface, facilitating the sharing of these free electrons with the d-orbitals of Fe atoms (supported by the red shift of the peaks associated with these groups) (Figure 15a,b).

## 4. Conclusions

In this study, the naturally occurring polysaccharide xanthan gum, commonly used as a thickening agent in the oil and gas industry, was examined as a potential active component against a saline CO_2_-saturated environment at both 30 °C and 90 °C. Based on the experimental results, the following conclusion can be drawn:

- XG proved to be an effective corrosion inhibitor against sweet corrosion, and its anticorrosive performance was found to increase with concentration but decrease with temperature. The maximum inhibition efficiency was found to be 70.10% and 61.41% at 30 °C and 90 °C, respectively, after 24 h of immersion.
- Long immersion time experiments showed that the IE% initially increases during the first hours up to 24 h, then slightly decreases with longer immersion times.
- PDP measurements have shown that XG functions as a mixed-type inhibitor, suppressing both cathodic and anodic reactions at both temperatures; however, at 90 °C, the suppression of the cathodic reaction was more dominant.
- The FTIR measurements reveal that XG was strongly adsorbed on the metal surface and that the adsorption process followed the Temkin adsorption isotherm. The adsorption and activation parameters indicated that the adsorption process occurred through both physical and chemical mechanisms.
- SEM confirmed the efficacy of XG as a corrosion inhibitor. Cross-section SEM analysis reveals a thicker corrosion layer when XG is absent at both temperatures.
- The favourable IE observed at high temperatures may be attributed to the helix/coil transition process, which increases the number and mobility of XG macromolecules available for absorption on the surface, thereby partially compensating for the weakened interactions at the molecules/metal interface.

## Figures and Tables

**Table 1:** Thermodynamic adsorption parameters of N80 carbon steel at 30 °C and 90 °C.

Temperature (°C)	*R* ^2^	*a*	*K*_ads_ (g L^−1^)	${∆G}_{\mathrm{ads}}^{{}^{\circ}}$ (kJ mol^−1^)
Langmuir				
30	0.989	-	3.60	−20.64
90	0.986	-	3.17	−21.66
Temkin				
30	0.989	−3.88	71.65	−28.18
90	0.960	−5.07	104.14	−29.12

**Table 2:** Apparent activation energies (*E*_a_) and heat of adsorption (*Q*_ads_) calculated in the temperature range of 30–90 °C.

*C*_inh_ (g L^−1^)	*E*_a_ (kJ mol^−1^)	*Q*_ads_ (kJ mol^−1^)
Blank	13.37	-
0.1	13.29	−2.17
0.5	15.81	−3.99
1.0	15.52	−4.06

**Table 3:** Potentiodynamic polarization parameters in the absence and presence of different concentrations of XG after 24 h of immersion time and at various temperatures.

*C*_inh_ (g L^−1^)	*β*_c_ (V dec^−1^)	*i*_corr_ (mA cm^−2^)	*E*_corr_ (V)	IE (%)
30 °C				
Blank	0.629 ± 0.051	0.607 ± 0.038	−0.711 ± 0.19	-
0.1	0.438 ± 0.028	0.441 ± 0.029	−0.712 ± 0.35	27.27
0.5	0.518 ± 0.035	0.224 ± 0.020	−0.710 ± 0.20	63.08
1.0	0.518 ± 0.015	0.160 ± 0.025	−0.727 ± 0.21	73.64
90 °C				
Blank	0.358 ± 0.011	1.505 ± 0.112	−0.708 ± 0.041	-
0.1	0.342 ± 0.027	1.069 ± 0.085	−0.734 ± 0.036	28.97
0.5	0.428 ± 0.018	0.819 ± 0.020	−0.727 ± 0.018	45.55
1.0	0.368 ± 0.010	0.508 ± 0.057	−0.724 ± 0.031	66.24

**Table 4:** EIS parameters in the absence and presence of different concentrations of XG after 24 h of immersion time, and at various temperatures.

*C*_inh_ (g L^−1^)	*R*_s_ (Ω cm^2^)	*CPE*_dl_		*R*_ct_ (Ω cm^2^)	*C*_dl_ (mF cm^−2^)	*L* (H cm^2^)	*R*_L_ (Ω cm^2^)	*C*_f_ (mF cm^−2^)	*R*_f_ (Ω cm^2^)	*R*_p_ (Ω cm^2^)	*χ*^2^ (×10^−3^)	IE (%)
		*Y*_dl_ (s^n^ mΩ^−1^ cm^−2^)	*n*_dl_									
30 °C												
Bank	20.64 ± 1.51	5.09 ± 1.12	0.744 ± 0.22	24.19 ± 2.22	19.81	-	-	0.71 ± 0.25	8.63 ± 2.11	32.82	0.37	-
0.1	19.56 ± 2.01	3.73 ± 0.91	0.896 ± 0.20	39.64 ± 4.31	3.74	-	-	0.98 ± 0.31	11.72 ± 2.22	51.36	0.99	36.10
0.5	23.37 ± 1.99	0.88 ± 0.54	0.938 ± 0.22	50.06 ± 7.50	1.04	-	-	0.63 ± 0.11	17.43 ± 3.44	67.49	3.64	51.37
1.0	24.45 ± 1.71	0.92 ± 0.47	0.906 ± 0.15	89.38 ± 6.21	1.24	-	-	0.74 ± 0.09	20.38 ± 2.37	109.76	2.72	70.10
90 °C												
Bank	10.75 ± 1.11	9.25 ± 2.58	0.781 ± 0.19	9.62 ± 1.19	27.98	-	-	1.28 ± 0.18	5.43 ± 2.01	15.05	0.22	-
0.1	10.51 ± 1.53	5.01 ± 1.41	0.832 ± 0.21	12.29 ± 1.29	9.84	-	-	2.09 ± 0.20	6.51 ± 1.11	18.80	0.92	19.95
0.5	10.69 ± 1.54	3.16 ± 1.11	0.872 ± 0.19	22.68 ± 3.15	5.00	1.38 ± 0.11	2.52 ± 0.95	1.94 ± 0.09	4.29 ± 1.89	29.49	0.28	48.96
1.0	10.89 ± 1.23	1.46 ± 1.01	0.891 ± 0.11	31.69 ± 4.11	1.97	4.96 ± 1.25	7.31 ± 1.19	-	-	39.00	0.77	61.41

## Data Availability

The original contributions presented in the study are included in the article, further inquiries can be directed to the corresponding author.

## Supplementary Materials

The following supporting information can be downloaded at: https://www.mdpi.com/article/10.3390/ma18194450/s1, Figure S1: The denaturation process of XG at high temperatures; Figure S2: Microstructure of the N80 pipeline steel sample; Table S1: Average corrosion rate and inhibition efficiency obtained from weight loss measurements at various concentrations of XG after 24 h of immersion at 30 and 90 °C; Table S2: Average corrosion rate at different immersion times and temperatures, before and after removing the corrosion products; Table S3: Comparison of the reported inhibition efficiency of XG and other natural corrosion inhibitors in different media with the present inhibitor; Table S4: EIS parameters in the absence and presence of 1.0 g L^−1^ of XG at 30 °C, and different immersion times; Table S5: EIS parameters in the absence and presence of 1.0 g L^−1^ of XG at 90 °C, and different immersion times; Table S6: EDS analysis carried out at different immersion times and temperatures.
