# Supplementary material for: Xanthan Gum as an Eco-Friendly Corrosion Inhibitor for N80 Carbon Steel Under High Pressure and High Temperature in Saline CO2-Saturated Solution
Source: Materials (Basel). 2025 Sep 23;18(19):4450. doi: 10.3390/ma18194450 (PMC12525378; doi:10.3390/ma18194450)
Supplement: Supplementary file 1 [file materials-18-04450-s001.zip › materials-3852774-supplementary.pdf]

Xanthan Gum as an Eco-Friendly Corrosion Inhibitor for N80 Carbon Steel Under High Pressure and High Temperature in Saline CO_2-Saturated Solution

Gaetano Palumbo

AGH University of Krakow, Faculty of Foundry Engineering, Department of Chemistry and Corrosion of Metals, al. A. Mickiewicza 30, 30-059 Krakow, Poland; gpalumbo@agh.edu.pl

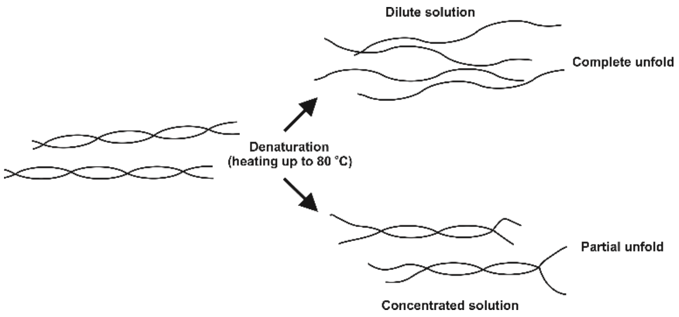

Figure S1. The denaturation process of XG at high temperatures

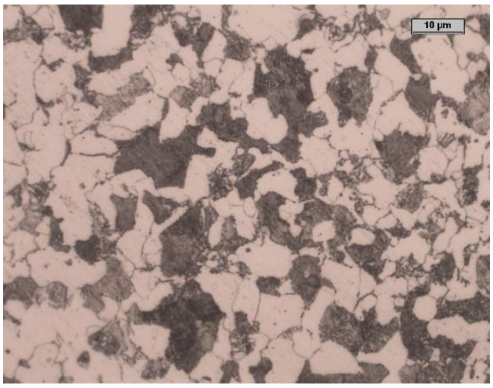

Figure S2. Microstructure of the N80 pipeline steel sample

Table S1. Average corrosion rate and inhibition efficiency obtained from weight loss measurements at various concentrations of XG after 24 hours of immersion at 30 and 90 °C

**C_{inh} (g L^{-1})**	**CR (mg cm^{-2} h^{-1})**	**IE (%)**	**CR (mg cm^{-2} h^{-1})**	**IE (%)**
	**30 °C**		**90 °C**	
Blank	0.470±0.058	-	1.105±0.151	-
0.1	0.341±0.065	26.67	0.815±0.120	23.24
0.5	0.272±0.064	41.51	0.691±0.091	37.47
1.0	0.211±0.053	55.11	0.556±0.067	49.68

Table S2. Average corrosion rate at different immersion times and temperatures, before and after removing the corrosion products

**C_{inh} (g L^{-1})**	**CR (mg cm^{-2} h^{-1})**	**IE (%)**	**CR (mg cm^{-2} h^{-1})**	**IE (%)**
	**30 °C**		**90 °C**	
		**24h**		
Blank (Before)	0.465±0.018	-	0.669±0.11	-
1.0 (Before)	0.201±0.053	56.77	0.442±0.083	33.93
Blank (After)	0.470±0.011	-	1.105±0.101	-
1.0 (After)	0.211±0.015	55.11	0.556±0.067	49.68
		**72h**		
Blank (Before)	0.650±0.46	-	0.755±0.100	-
1.0 (Before)	0.388±0.013	40.31	0.580±0.045	23.18
Blank (After)	0.811±0.105	-	1.151±0.123	-
1.0 (After)	0.452±0.025	44.27	0.700±0.101	39.18

Table S3. Comparison of the reported inhibition efficiency of XG and other natural corrosion inhibitors in different media with the present inhibitor

**Inhibitors**	**Metal**	**Corrosive media**	**Inhibitor concentration (g L^{-1})**	**Temperature (°C)**	**IE (%)**	**Time of exposure (h)**	**Reference**
Xanthan gum	Low Carbon Steel	1 M HCl	1	30/60	74.24/55.40	6	[1]
Xanthan gum	Carbon Steel (L80)	1 M HCl	0.2	25	72.72	12	[2]
Xanthan gum	Carbon Steel (Q235)	1 M HCl	0.8	30	78.66	6	[3]
Xanthan gum	Carbon Steel	15% HCl	0.4	25/60	90.20/60.57	6	[4]
Xanthan gum	Carbon Steel (X80)	1 M H_2SO_4	0.2	20	83.54		[5]
Carboxymethyl cellulose	API 5L X60 Steel	3.5% NaCl in CO_2	1.5	25	39	1	[6]
Chitosan	API 5L X60 Steel	3.5% NaCl in CO_2	1	25	45	1	[6]
Guar gum	Carbon Steel (N80)	0.5 M KCl in CO_2	0.4	25/50	74.55/63.13	24	[7]
Gum arabic	Carbon Steel (N80)	0.5 M KCl in CO_2	0.5	25/45	68.78/40.74	24	[8]
Xanthan gum	Carbon Steel (N80)	0.5 M KCl in CO_2	1.0	30/90	55.11/49.68	24	Present work
Xanthan gum	Carbon Steel (N80)	0.5 M KCl in CO_2	1.0	30/90	44.27/28.97	72	Present work

Table S4. EIS parameters in the absence and presence of 1.0 g L^{-1} of XG at 30 °C, and different immersion times

**Time (h)**	R_s **(Ω cm²)**	CPE_{dl}		R_{ct} **(Ω cm²)**	L **(H cm²)**	R_L **(Ω cm²)**	C_f **(F cm⁻²)**	R_f **(Ω cm²)**	R_p **(Ω cm²)**	χ^2 **(× 10⁻³)**	**IE (%)**
		Y_{dl} **(s^n mΩ⁻¹ cm⁻²)**	n_{dl}								
					**Blank (30 °C)**						
6	18.35	1.36	0.833	48.40	-	-	1.10	6.78	55.18	0.49	-
12	19.42	1.64	0.804	31.31	-	-	0.95	7.27	38.58	0.31	-
24	20.64	5.09	0.744	24.19	-	-	0.71	8.63	32.82	0.37	-
48	20.82	6.56	0.774	20.83	-	-	0.79	8.96	29.76	0.14	-
72	19.76	9.28	0.787	19.55	-	-	1.01	7.71	27.26	0.45	-
					**XG (30 °C)**						
6	20.35	0.61	0.856	106.7	-	-	0.68	18.22	124.92	1.80	55.83
12	23.17	0.75	0.895	90.72	-	-	0.75	20.01	110.73	2.89	65.16
24	24.67	0.92	0.906	89.38	-	-	0.74	20.38	109.76	2.72	70.10
48	24.45	1.11	0.896	69.21	-	-	0.54	24.38	93.59	2.13	68.20
72	20.86	2.34	0.914	38.29	-	-	0.91	19.04	57.33	0.96	52.45

Table S5. EIS parameters in the absence and presence of 1.0 g L^{-1} of XG at 90 °C, and different immersion times

**Time (h)**	R_s **(Ω cm²)**	CPE_{dl}		R_{ct} **(Ω cm²)**	L **(H cm²)**	R_L **(Ω cm²)**	C_f **(F cm⁻²)**	R_f **(Ω cm²)**	R_p **(Ω cm²)**	χ^2 **(× 10⁻³)**	**IE (%)**
		Y_{dl} **(s^n mΩ⁻¹ cm⁻²)**	n_{dl}								
					**Blank (90 °C)**						
6	11.16	2.27	0.890	12.20	-	-	0.85	3.29	15.49	0.43	-
12	11.10	7.87	0.871	10.56	-	-	0.12	4.74	14.42	0.31	-
24	10.75	9.25	0.781	9.62	-	-	1.28	5.43	15.05	0.22	-
48	9.91	10.38	0.790	10.17	-	-	2.00	6.07	16.24	0.22	-
72	10.93	9.51	0.792	10.21	-	-	2.01	5.11	15.32	0.16	-
					**XG (90 °C)**						
6	10.97	0.47	0.900	19.17	1.82	8.11	-	-	27.28	2.54	43.22
12	10.52	1.03	0.890	21.36	2.77	8.12	-	-	29.48	1.86	51.09
24	10.89	1.46	0.891	31.69	4.96	7.31	-	-	39.00	0.77	61.41
48	10.57	2.08	0.896	24.03	6.86	8.38	-	-	32.41	0.68	49.89
72	10.01	3.37	0.855	20.43	3.06	5.41	-	-	25.84	0.69	40.71

Table S6. EDS analysis carried out at different immersion times and temperatures

**Element (wt.%)**	**C**	**O**	**Si**	**V**	**Mn**	**Cu**	**Fe**	**Cl**	**K**	**Total**
Polished	0.39	-	0.26	0.19	1.80	0.26	97.14	-	-	100
30 °C										
Blank (24h)	6.0	1.5	0.2	0.2	1.5	0.9	89.3	0.2	0.2	
XG 1.0 g/L (24h)	2.7	0.8	0.2	0.1	0.5	-	95.7	-	-	100
Blank (72h) (1)	5.7	33	0.2	-	0.9	-	59.2	0.8	0.2	100
Blank (72h) (2)	5.0	1.9	0.2	0.3	1.5	0.7	90.4	-	-	100
XG 1.0 g/L (72h) (1)	5.9	13.0	0.1	1.6	4.3	3.2	70.9	0.1	0.9	100
XG 1.0 g/L (72h) (2)	4.1	1.2	0.3	0.2	1.7	0.1	92.3	0.1	-	100
90 °C										
Blank (24h) (1)	14.2	29.6	-	-	1.1	-	56.2	-	-	100
Blank (24h) (2)	24.8	21.1			0.4		15.8	18.2	19.7	100
XG 1.0 g/L (24h) (1)	22.3	38.1	-	-	0.8	-	38.8	-	-	100

XG 1.0 g/L (24h) (2)	44.9	10.0	-	0.7	1.9	1.7	40.2	0.3	0.3	100
Blank (72h) (1)	9.7	25.6	-	-	0.7	-	62.7	0.6	0.7	100
Blank (72h) (2)	3.8	6.8	-	-	1.2	0.2	87.5	0.2	0.3	100
XG 1.0 g/L (72h) (1)	13.6	43.2	-	-	0.7	-	42.5	-	-	100
XG 1.0 g/L (72h) (2)	8.9	8.5	-	-	-	-	4.4	36.3	41.9	100

The impedance of a *CPE* is described by Eq. S1, whereby Y_o is the double layer *CPE* coefficient, j is an imaginary number ($j = (-1)^{\frac{1}{2}}$, ω is the angular frequency in rad/s, and n (with value $-1 \leq n \leq 1$) is the phase shift, which also indicates the homogeneity (smoothness) of the corroding surface [9]. The double layer capacitance (C_{dl}) can be calculated as a function of R_s, R_{ct}, Y_o, and n, based on the model proposed by Brug et al. [10], according to Eq S2.

$$Z_{CPE} = Y_o[(j\omega)^n]^{-1} \quad \text{(S1)}$$

$$C_{dl} = Y_o^{\frac{1}{n}} \left[\frac{1}{R_s} + \frac{1}{R_{ct}}\right]^{\frac{n-1}{n}} \quad \text{(S2)}$$

$$C_{dl} = \frac{\varepsilon_0 \varepsilon A}{\delta} \quad \text{(S3)}$$

Where ε is the dielectric constant of the medium, ε_o is the vacuum permittivity, A is the electrode area, and δ is the thickness of the protective layer [8].

References

1. Mobin, M.; Rizvi, M. Inhibitory effect of xanthan gum and synergistic surfactant additives for mild steel corrosion in 1M HCl. *Carbohydr. Polym.* **2016**, *136*, 384-393, https://doi.org/10.1016/j.carbpol.2015.09.027.
2. Cao, Y.; Zou, C.; Wang, C.; Liang, H.; Lin, S.; Liao, Y.; Shi, L. β-cyclodextrin modified xanthan gum as an eco-friendly corrosion inhibitor for L80 steel in 1 M HCl. *Cellulose* **2021**, *28*, 11133-11152, https://doi.org/10.1007/s10570-021-04240-8.
3. Fu, W.; Sun, L.; Zhang, X.; Xu, H.; Cao, M. Enhancing metal corrosion inhibition with xanthan gum: The synergistic role of anionic surfactants. *Colloids Surf. A.* **2025**, *711*, 136364, https://doi.org/10.1016/j.colsurfa.2025.136364.
4. Biswas, A.; Pal, S.; Udayabhanu, G. Experimental and theoretical studies of xanthan gum and its graft co-polymer as corrosion inhibitor for mild steel in 15% HCl. *Appl. Surf. Sci.* **2015**, *353*, 173-183, https://doi.org/10.1016/j.apsusc.2015.06.128.
5. Cao, Y.; Zou, C.; Wang, C.; Chen, W.; Liang, H.; Lin, S. Green corrosion inhibitor of β-cyclodextrin modified xanthan gum for X80 steel in 1 M H2SO4 at different temperature. *Journal of Molecular Liquids* **2021**, *341*, 117391, https://doi.org/10.1016/j.molliq.2021.117391.
6. Umoren, S.A.; AlAhmary, A.A.; Gasem, Z.M.; Solomon, M.M. Evaluation of chitosan and carboxymethyl cellulose as ecofriendly corrosion inhibitors for steel. *Int. J. Biol. Macromol.* **2018**, *117*, 1017-1028, https://doi.org/10.1016/j.ijbiomac.2018.06.014.
7. Palumbo, G.; Święch, D.; Górny, M. Guar Gum as an Eco-Friendly Corrosion Inhibitor for N80 Carbon Steel under Sweet Environment in Saline Solution: Electrochemical, Surface, and Spectroscopic Studies. *International Journal of Molecular Sciences* **2023**, *24*, 12269.
8. Palumbo, G.; Górny, M.; Banaś, J. Corrosion Inhibition of Pipeline Carbon Steel (N80) in CO2-Saturated Chloride (0.5 M of KCl) Solution Using Gum Arabic as a Possible Environmentally Friendly Corrosion Inhibitor for Shale Gas Industry. *J. Mater. Eng. Perform.* **2019**, *28*, 6458-6470, https://doi.org/10.1007/s11665-019-04379-3.
9. Obot, I.B.; Onyeachu, I.B.; Umoren, S.A. Alternative corrosion inhibitor formulation for carbon steel in CO2-saturated brine solution under high turbulent flow condition for use in oil and gas transportation pipelines. *Corros. Sci.* **2019**, *159*, 108140, https://doi.org/10.1016/j.corsci.2019.108140.

10. Brug, G.J.; van den Eeden, A.L.G.; Sluyters-Rehbach, M.; Sluyters, J.H. The analysis of electrode impedances complicated by the presence of a constant phase element. *Journal of Electroanalytical Chemistry and Interfacial Electrochemistry* **1984**, *176*, 275-295, https://doi.org/10.1016/S0022-0728(84)80324-1.
